# Deep learning-based automated segmentation for the quantitative diagnosis of cerebral small vessel disease via multisequence MRI

**DOI:** 10.3389/fneur.2025.1540923

**Published:** 2025-05-27

**Authors:** Huiyu Zhao, Miaoyi Zhang, Weijun Tang, Luyuan Jin, Jie Tang, Langfeng Shi, Xiao Deng, Jianhui Fu, Weiwen Zou

**Affiliations:** ^1^The State Key Laboratory of Advanced Optical Communication Systems and Networks, Intelligent Microwave Lightwave Integration Innovation Center (imLic), Department of Electronic Engineering, Shanghai Jiao Tong University, Shanghai, China; ^2^Department of Neurology, Huashan Hospital, Fudan University, Shanghai, China; ^3^Department of Radiology, Huashan Hospital, Fudan University, Shanghai, China

**Keywords:** cerebral small vessel diseases, deep learning, global burden of disease, quantitative evaluation, multisequence MRI

## Abstract

**Objective:**

Existing visual scoring systems for cerebral small vessel disease (CSVD) cannot assess the global lesion load accurately and quantitatively. We aimed to develop an automated segmentation method based on deep learning (DL) to quantify the typical neuroimaging markers of CSVD on multisequence magnetic resonance imaging (MRI).

**Materials and methods:**

MRI scans from internal (July 2018 to July 2022) and external (November 2012 to January 2015) datasets were analyzed. A DL-based segmentation method was developed to evaluate the quantitative volumes of white matter hyperintensity (WMH), cerebral microbleeds (CMBs), lacunes, and enlarged perivascular spaces (EPVSs) according to the segmentation results. Dice and other quantitative metrics were used to access the DL segmentation results. Pearson correlation coefficients were used for correlation analysis, and the differences in marker volumes among different visual scores were assessed via analysis of variance (ANOVA). Finally, a quantitative Z score was calculated to represent CSVD-related brain burden.

**Results:**

A total of 105 internal patients (64.8 ± 7.4 years, 70 males) and 58 external patients (68.2 ± 6.8 years, 29 males) were evaluated. The Dice values for WMH, CMBs, lacunes, and EPVSs in the internal dataset were 0.85, 0.74, 0.76, and 0.75, respectively. The positive correlation between the DL and the manual approach results was excellent (overall Pearson correlation = 0.968, 0.978, 0.948, and 0.947, respectively). The predicted volumes of the CSVD neuroimaging markers showed significant differences among the groups with different visual scores (*p* < 0.001). The quantitative Z scores reflecting CSVD global burden also correlated well with the widely recognized total burden score (*p* < 0.001).

**Conclusion:**

An automated DL model was developed for the segmentation of four CSVD neuroimaging markers on multisequence MRI, providing a strong basis for further CSVD research.

## 1 Introduction

Cerebral small vessel disease (CSVD) is a group of pathological processes that affect the small arteries, arterioles, capillaries, and venules of the brain. CSVD can cause ischemic stroke, cognitive decline, neurobehavioral symptoms, and functional impairment, posing a significant public health threat to the elderly (1). Since CSVD often occurs and develops insidiously (2), magnetic resonance imaging (MRI) is widely employed to detect and diagnose CSVD. Common neuroimaging markers of CSVD include white matter hyperintensity (WMH), lacunes, cerebral microbleeds (CMBs), and enlarged perivascular spaces (EPVSs) (3), which are variably associated with the clinical performance and progression of CSVD. The potential mechanisms underlying CSVD include chronic cerebral ischemia, hypoperfusion, endothelial dysfunction, blood–brain barrier disruption, glymphatic dysfunction, and inflammatory responses (4). Although different CSVD markers result from diverse pathophysiologic processes, they may manifest simultaneously in the brain (5). Notably, recent longitudinal studies have demonstrated that the combined quantification of multiple markers provides stronger predictive value for clinical outcomes than individual markers (6, 7). Thus, emphasizing the overall impact of CSVD on the brain is more meaningful than considering individual markers in isolation.

Visual scoring systems that combine multiple neuroimaging features, including 4- and 6-point rating scores, have been developed to qualitatively represent the total imaging burden of CSVD (4). While the qualitative tools may achieve better generalizability, the inevitable limitations are as follows: First, scale complexity may have an adverse effect on interrater reliability, especially in population-level studies (8). Second, the accuracy of neuroimaging diagnosis based on visual rating scores cannot be guaranteed due to variations in clinician expertise. Third, when used for the analysis of larger datasets, obtaining qualitative scores is a time-consuming and labor-intensive process. Further, as the impact of CSVD on the brain is a dynamic process that continuously changes over time (9), qualitative scores cannot be used to measure the global lesion load accurately and quantitatively. Therefore, a quantitative tool is highly needed for the precise and rapid diagnosis of CSVD.

In the last decade, extensive advances have been made using deep learning (DL) for medical image processing because of its advantages in accuracy, efficiency, and repeatability (10–12). Several DL models have recently been developed for segmentation and detection in the CSVD field (13, 14). Among the typical CSVD neuroimaging markers, WMH has received more attention than others (15–20). For example, a network called DeepWML was proposed in Zhang et al. (16) for automated detection and segmentation of WMH lesions in MRI images. Other lesions like CMBs and lacune have also garnered considerable research interest (21, 22). However, there remains a lack of consensus on procedures for segmenting and quantifying all CSVD neuroimaging markers. The segmentation of EPVSs, in particular, remains a significant challenge because of the time-consuming manual delineation and the difficulties in identification. In addition, the multiple lesions in CSVD have different imaging characteristics, which prevents general DL methods from accurate segmentation (23). Using separate DL models for each marker would also result in a large increase in the data required for each marker, and the similarity of the different sequences is not well utilized. Thus, the multi-marker-adapted DL model of CSVD requires further exploration.

In this study, we aimed to develop a fully automated, highly accurate algorithm for multiple markers segmentation that can be used to detect the four typical neuroimaging markers of CSVD over multiple sequences of brain MRI. Then, a “share learning” strategy and cross-sequence attention mechanisms were proposed to leverage anatomical consistency across modalities, overcoming limitations of prior single-sequence approaches. Thirdly, a quantitative segmentation-based total CSVD burden score was generated from the proposed DL method that correlates with established clinical scales while enabling millimeter-level volumetric precision. From a clinical perspective, the proposed framework transformed the current qualitative CSVD assessments to quantitative diagnostics, providing clinicians with an objective tool for monitoring CSVD progression and enabling personalized risk stratification, thereby establishing a robust foundation for more precise and individualized diagnoses in the future.

## 2 Materials and methods

### 2.1 Ethics statement

The current study conformed with the World Medical Association Declaration of Helsinki and was approved by the Research Ethics Committee of Huashan Hospital (Project ID:KY2018–224). All of the participants or their relatives provided written informed consent.

### 2.2 Patient datasets

**Figure 1:**
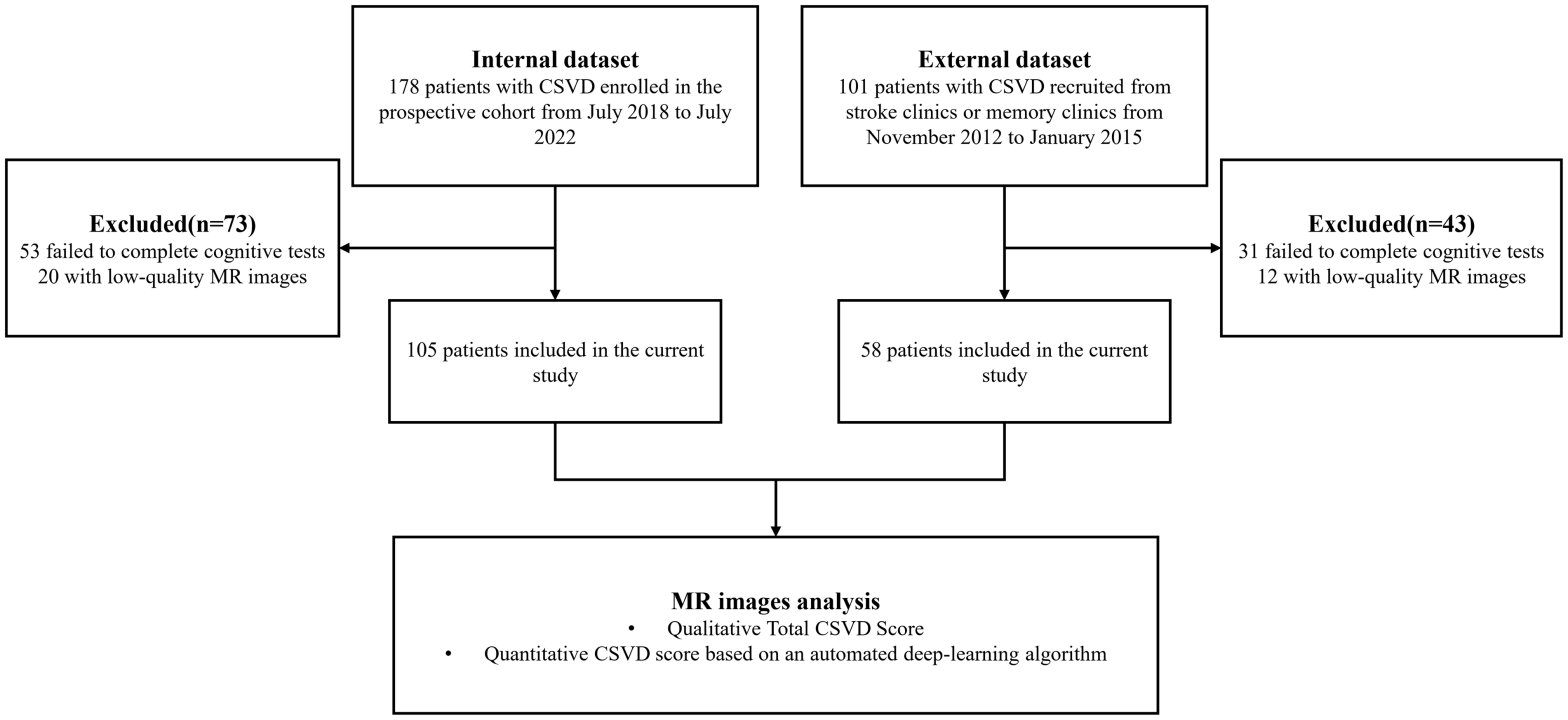
The flowchart of participant inclusion processes in the internal and external datasets. CSVD, cerebral small vessel disease; MR, magnetic resonance; DL, deep learning.

To develop the DL model, we used an internal dataset of 178 patients with arteriosclerotic CSVD prospectively enrolled from July 2018 to July 2022 at North Huashan Hospital (registration number: ChiCTR1800017902). The algorithm was then tested on an external dataset of 101 patients recruited from stroke clinics or memory clinics at Huashan Hospital from November 2012 to January 2015. The full inclusion and exclusion criteria for the two datasets have been published previously (24, 25) and are listed in Supplementary material. Patients with low-quality images were further excluded. The details of the inclusion process are shown in Figure 1. Demographic characteristics and vascular risk information were also collected.

### 2.3 Imaging protocol

All of the patients in the internal dataset were scanned via a 3-T MRI scanner (GE HDxt 3.0 T, scanner software version: HD16.0_V02_1131). The imaging protocol included three-dimensional magnetization prepared rapid acquisition gradient echo T1-weighted imaging (3D-MPRAGE T1WI), T2-weighted imaging (T2WI), fluid-attenuated inversion recovery (FLAIR) imaging, and susceptibility-weighted angiography (SWAN). The 3D-MPRAGE parameters were as follows: repetition time (TR) = 9.7 ms, echo time (TE) = 3.0 ms, flip angle = 15°, slice thickness = 1 mm, field of view (FOV) = 256 mm, matrix = 256 × 256, and voxel size = 1 mm × mm × 1 mm. The T2WI parameters were TR = 3,620 ms, TE = 120 ms, slice thickness = 6 mm, and FOV = 240 mm. The FLAIR parameters were TR = 9,675 ms, TE = 150 ms, slice thickness = 2 mm, FOV = 240 mm, and matrix = 480 × 480. The SWAN parameters were TR = 62.1 ms, TE = 32 ms, flip angle = 15°, slice thickness = 1.6 mm, FOV = 240 mm, and matrix = 240 × 240. When performing the SWAN sequence scanning, a two-fold phase acceleration was obtained using the parallel acquisition technique.

All of the patients in the external validation dataset were scanned via a 3 Tesla scanner (Siemens Magneton Verio3T). The MRI sequences included 3D-MPRAGE T1WI, T2WI, FLAIR, and SWI. The 3D-MPRAGE parameters were: TR = 2,300 ms, TE = 2.98 ms, flip angle = 9°, slice thickness = 1.0 mm, FOV = 256 mm, matrix = 256 × 256, voxel size = 1 mm × 1 mm × 1 mm. The T2WI parameters were: TR = 3,500 ms, TE = 95 ms, FOV = 200 mm × 230 mm, slice thickness = 6 mm, matrix = 256 × 256. The FLAIR were: TR = 9,000 ms, TE = 102 ms, FOV = 200 mm × 230 mm, slice thickness = 6 mm, matrix = 256 × 190. The SWI parameters were: TR = 28 ms, TE = 20 ms, flip angle = 15°, slice thickness = 1.2 mm, FOV = 172 mm × 230 mm, and matrix = 221 × 320.

### 2.4 Manual annotation

WMH, CMBs, lacunes, and EPVSs were determined on the basis of the STandards for ReportIng Vascular changes on nEuroimaging 2 (STRIVE-2) (3). In this study, WMH was graded according to the sum of deep and periventricular WMH Fazekas scales (0 to 3): 1 = total periventricular+ subcortical WMH grade 3–4; 2 = grade 5–6. The numbers of CMBs and lacunes were respectively recorded >4 lacunes. Basal ganglia, centrum semiovale, and midbrain regions are reported as three major sites for EPVSs (26). The existing total burden score puts more emphasis on the number of EPVSs in the basal ganglia (6). Thus, the categories of EPVSs used in the current study considered EPVSs in the basal ganglia as follows: 0 = none, 1 = 1–10, 2 = 11–20, 3 = 21–40, and 4 = 40 (26). We rated the total CSVD burden on an ordinal scale from 0 to 4, as previously reported (6). The ground truth segmentations were delineated by three experienced clinicians (with 7, 5, and 3 years of neuroimaging expertise) blinded to the clinical data and group information for each examination. To quantify inter-rater variability, we calculated the kappa value, Dice similarity coefficient (Dice), and intraclass correlation coefficient (ICC) for randomly selected 50% of the dataset. All of the segmentation results were subsequently reviewed by a senior radiologist (28 years of experience), with discrepancies resolved through consensus discussion.

### 2.5 Image preprocessing

Image preprocessing included format conversion, size and value normalization, and positive sample data augmentation. The MRI data for all three sequences were in *dcm* format. The raw data were manually segmented via itk-snap 3.8.0 and the labels were saved as *nii* files. The SimpleITK and Nibabel libraries were subsequently used to read and save the *dcm* and *nii* data into *npy* format with the same matrix size, respectively. Then, all of the images were resized to 320 × 320 and normalized to [0–1] via min-max normalization. To address class imbalance in the MRI dataset where positive samples were underrepresented, we implemented comprehensive data augmentation to prevent model bias toward negative predictions. Our augmentation strategy included the following methods: (1) geometric transformations (vertical/horizontal flipping, ±30° rotation); (2) spatial adjustments (x/y-axis displacement up to 20% of image dimensions); and (3) scale variations (0.8–1.2 × resizing). The data augmentation techniques expanded the positive sample size by a factor of 11, which significantly improved network performance by balancing class distribution while preserving lesion characteristics.

### 2.6 Deep learning model

**Figure 2:**
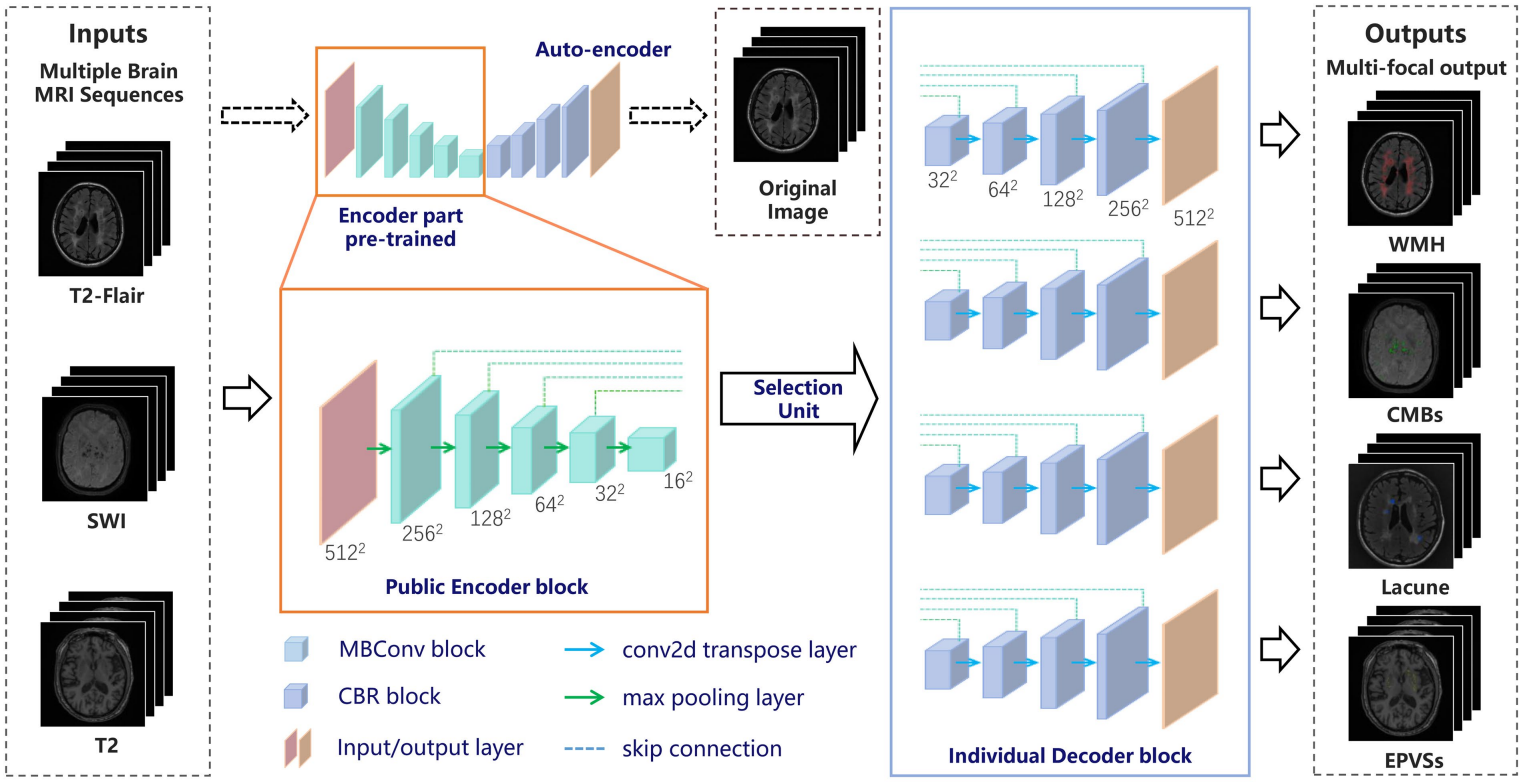
The overall framework of the proposed network. MRI, magnetic resonance imaging; CSVD, cerebral small vessel disease; WMH, white matter hyperintensity; CMBs, cerebral microbleeds; EPVSs, enlarged perivascular spaces.

A DL model was developed to collect images from three different sequences (3D-MPRAGE T1WI, FLAIR, and SWAN/SWI) and output their corresponding segmentation results for each of the four markers. Figure 2 shows the proposed DL framework, which consists of two parts: (a) an auto-encoder network used to pretrain the encoder layers of the subsequent segmentation network and (b) a multi-output U-shaped network, named MO-UNET, used to segment different markers.

#### 2.6.1 Pretraining with the autoencoder

First, the autoencoder, which consists of an encoder and a decoder, was trained using the original MRI data. The encoder extracts useful features from high-dimensional input data and maps them into a low-dimensional space. The decoder then recovers the input data from the low-dimensional vectors via transposed convolution. Autoencoder is able to extract local features of images and apply these features to further tasks such as target detection, image segmentation, and image reconstruction. Here we used EfficientNet (27) as the encoder part, enabling richer feature representations. Additionally, the parameters of EfficientNet have been pretrained and tuned to be highly generalizable and robust to migration learning on various datasets and tasks.

#### 2.6.2 The proposed network

To address the dual challenges of cross-sequence feature sharing and marker-specific differentiation in CSVD MRI analysis, we propose a Multi-Output UNet (MO-UNet) architecture that synergizes multi-task learning with sequence-aware adaptation. Built upon the U-Net framework (28), MO-UNet employs a shared EfficientNet-based encoder pre-trained via contrastive autoencoding to extract fundamental vascular patterns common across MRI sequences (T1/T2-FLAIR/SWI), followed by four dedicated decoders that preserve marker-specific characteristics. Specifically, the encoder utilizes stacked MBConv blocks, namely Mobile Inverted Residual Bottleneck blocks, with each comprising the following components: (1) a depthwise separable convolution layer and a 1 × 1 pointwise convolution layer that is used to reduce the number of model parameters and computational complexity; (2) an adaptive activation function called the “Swish” activation function to better address gradient vanishing and gradient explosion; and (3) a residual connection that helps information transfer and gradient propagation. These designs allow the model to reduce the computational complexity and the number of parameters while maintaining a high level of accuracy, improving the computational efficiency of the network. The encoder is designed to be public so that all slices must pass through the shared encoder before entering the selection unit. Thus, the network has the ability to preserve the similarity of each sequence in the encoding phase. Then, at the encoder-decoder interface, a dynamic selection unit routes features to target decoders based on input sequence type.

The decoder part of the designed network has four decoder modules, representing four clinical markers of CSVD. Each module restores the encoder feature maps to the spatial resolution of the original image. This design allows the network to preserve the specificity of different CSVD markers in the decoding phase. Like the conventional U-shaped structure, each decoder module is merged with the encoder feature map at different scales through a skip connection, and the features are progressively recovered through the upsampling and convolution layers. Each decoder integrates spatial-channel squeeze & excitation (scSE) blocks (29) as the attention mechanism to increase the accuracy and efficiency of segmentation. The spatial attention gates suppress irrelevant backgrounds, while channel attention amplifies marker-specific frequency components. By co-training all decoders on mixed sequences, the model learns both shared vascular representations through encoder and marker-specific boundaries through decoders. At last, each decoder outputs the segmentation results for the corresponding CSVD markers.

Overall, to fit both the similarity and specificity characteristics of CSVD with multiple sequences and multiple markers, a “shared learning” concept is proposed in which all of the images are input into the same encoding phase. This strategy greatly avoids overfitting or underfitting caused by insufficient data, especially in the medical field. In the decoding phase, by using the selection unit and multiple decoding modules, the different CSVD marker segmentation results corresponding to each sequence are obtained by multiple output channels. This step preserves the specific structural features of different CSVD markers, which is particularly useful when confronted with different MRI sequences that have both similarities and specificities. The overall architecture improves the accuracy of the segmentation task, enabling researchers to perform more precise analysis and diagnosis.

#### 2.6.3 Loss function

The network combines cross-entropy and Dice loss to form the loss function. Cross-entropy loss is widely used for binary classification of pixel points, which can be expressed as follows:

$$L_{\mathrm{ce}}=-[\text{ylog}\;(\overset{⌣}{y})+(1-y)\log(1-\overset{⌣}{y})]$$

where y represents the true label and $\overset{⌣}{y}$ represents the predicted label of each pixel. Dice loss is another commonly used loss function for segmentation tasks, which can be expressed as follows:

$$L_{\text{Dice}}=1-\frac{2|X\cap Y|}{|X|+|Y|}$$

where X is the set of real labels and Y is the set of predicted labels. The cross-entropy loss calculates all of the pixels of an image with no difference between the foreground and background pixels, which may incline the model to predict all of the pixels as background pixels, leading to training failure. Dice loss excludes the negative influence of background pixels during training, which helps solve the problem of positive and negative sample imbalance. However, using Dice loss alone may not provide enough gradient information to guide model learning, especially in the initialization phase. Thus, we use the combination of the cross-entropy and Dice loss as the proposed loss function:

$$L=L_{\mathrm{ce}}+L_{\text{Dice}}$$

### 2.7 Experimental settings

Multisequence MR images from 105 patients were used as internal data for developing DL algorithms. The dataset was partitioned at the patient level to ensure independence between training and validation sets. To rigorously optimize hyperparameters and evaluate model generalizability, we performed five-fold nested cross-validation on the internal cohort. Specifically, the full dataset was divided into five patient-stratified folds (21 patients/fold), with each fold iteratively held out as the test set. For the remaining 84 patients in each iteration, an 80:20 train-validation split was applied to tune hyperparameters. This process was repeated across all five folds. Then, the best parameters were selected based on maximum validation accuracy for the subsequent independent test set of 58 patients. During training, an Adam optimizer (30) with L2 weight decay (*λ* = 0.0001) was used because of its fast convergence and high computational efficiency. The learning rate varies during training according to the following formula:

$$lr_{\text{decayed}}=lr_{\text{initial}}*\mathrm{rat}e^{\frac{\mathrm{ste}p_{\text{global}}}{\mathrm{ste}p_{\text{decay}}}}$$

where $lr_{\text{initial}}=0.001$ is the initial learning rate, and $lr_{\text{decayed}}=0.95$ indicates the decay rate. $\mathrm{ste}p_{\text{global}}$ is a global variable representing the current number of iteration rounds of training, and $\mathrm{ste}p_{\text{decay}}=500$ is the set decay period. Training continued for 100 epochs and terminated until no further improvement was achieved in five consecutive rounds. The batch size was set to 6 and the hyperparameters were set to epsilon = 0.001 and momentum = 0.99. Three DL models: UNET (29), Res-Net (31), and DeepLabV3 (32) were chosen as the comparison methods. All methods utilized the same datasets and experimental settings to ensure fairness. The algorithms were developed in-house via Python version 3.6.5,^1^ implemented using PyTorch version 1.8.1,^2^ and trained with two NVIDIA GeForce RTX 3090 GPUs. The code is available at https://github.com/HYZimLic/MO-UNET.

### 2.8 Statistical analysis

Statistical analyses were performed via SPSS 26.0 software. Inter- and intra-rater agreement measurements for the total burden score and ground truth were evaluated with kappa values. To assess intra-rater reliability, each clinician assessed the images of all patients twice, with a 6-month interval between assessments. Categorical variables are presented as counts and percentages. Continuous variables are presented as the means [standard deviations (SDs)].

The precision (Pre), specificity (Sp), and Dice coefficient of the proposed DL framework are calculated based on the segmentation masks of all four CSVD markers in comparison with the labels of physicians. All of the results are obtained from the confusion matrices corresponding to true positive (TP), true negative (TN), false positive (FP), and false negative (FN) results. The formulas are as follows:

$$\mathrm{Pre}=\frac{\mathrm{TP}}{\mathrm{TP}+\mathrm{FP}}$$

$$\mathrm{Sp}=\frac{\mathrm{TN}}{\mathrm{FP}+\mathrm{TN}}$$

$$\text{Dice}=\frac{2\mathrm{TP}}{\mathrm{FP}+2\mathrm{TP}+\mathrm{FN}}$$

The Hausdorff distance (HD95) was also being tested between different methods and the gold standard. HD95 is used to compute the 95th percentile of the distance between two point sets and is applied to the distance metric between two 3D image voxels. Given two masks X and Y , the HD 95 is calculated as follows:

HD95(X,Y)=max[d(X,Y),d(Y,X)]×95%

where d(⋅) represents the minimum Euclidean distance. For lesion-level analysis, we considered an area covering at least 50% of the lesion as a true positive and used this to calculate the per-lesion sensitivity (Sen):

$$\mathrm{Sen}=\frac{TP_{\text{lesion}}}{TP_{\text{lesion}}+FN_{\text{lesion}}}$$

In addition, the correlation between the quantitative volume of the CSVD markers obtained via DL and that labeled by clinicians was also evaluated via the Pearson correlation coefficient and Bland–Altman analysis. The formula for calculating the Pearson coefficient is as follows:

$$r=\frac{\sum (x_{i}-\bar{x})(y_{i}-\bar{y})}{\sqrt{\sum {\left(x_{i}-\bar{x}\right)}^{2}{\left(y_{i}-\bar{y}\right)}^{2}}}$$

where $x_{i}$ and $y_{i}$ represent the values of the x- and y-variables in a sample, respectively, while $\bar{x}$ and $\bar{y}$ represent the mean of the values of the x- and y-variables. The differences in CSVD marker volumes among different visual scores were assessed via analysis of variance (ANOVA). Since the volume of each CSVD marker varies, we then calculated the Z score of each CSVD marker and summed the four scores to obtain a quantitative result of the CSVD-related brain burden. The least significant difference (LSD) test was used for multiple comparisons, assuming equal variances.

## 3 Results

### 3.1 Characteristics of patient datasets

The current study included a total of 105 patients in the internal dataset (64.8 ± 7.4 years, 70 males and 35 females) and 58 subjects in the external dataset external dataset (68.2 ± 6.8 years, 29 males and 26 females). Table 1 summarizes the demographics and characteristics of the participants.

**Table 1:** The demographics and characteristics of the participants in the internal and external datasets.

Characteristics	Internal dataset (*n* = 105)	External dataset (*n* = 58)
Sex (male/female)	70/35	29/26
Age, year, mean (SD)	64.78 ± 7.41	68.16 ± 6.84
Education, year, mean (SD)	8.67 ± 4.51	11.51 ± 3.85
Hypertension, *n* (%)	94 (89.52)	46 (79.31)
Diabetes mellitus, *n* (%)	25 (23.81)	12 (20.69)
Hyperlipidemia, *n* (%)	32 (30.47)	25 (43.10)
History of smoking, *n* (%)	19 (18.10)	14 (24.14)
History of drinking, *n* (%)	11 (10.48)	11 (18.97)
WMH score, *n* (%)		
0	1 (0.95)	9 (15.52)
1	33 (31.43)	15 (25.86)
2	71 (67.62)	34 (65.38)
Number of CMBs, *n* (%)		
0	20 (19.05)	27 (46.55)
1–10	28 (26.67)	17 (29.31)
>10	57 (54.29)	14 (24.14)
Number of lacunes, *n* (%)		
0	29 (27.62)	36 (62.07)
1–4	43 (40.95)	18 (31.03)
>4	33 (31.43)	4 (6.90)
EPVSs score in basal ganglia, *n* (%)		
1	20 (19.05)	18 (31.03)
2	37 (35.24)	19 (32.76)
3	28 (26.67)	17 (29.31)
4	20 (19.05)	4 (6.90)
Total CSVD score(0–4), *n* (%)		
1	14 (13.33)	15 (25.86)
2	15 (14.29)	19 (32.76)
3	41 (39.05)	15 (25.86)
4	35 (33.33)	9 (15.52)

SD, standard deviation; WMH, white matter hyperintensity; CMBs, cerebral microbleeds; EPVSs, enlarged perivascular spaces; CSVD, cerebral small vessel disease. ^*^The total CSVD score ranges from 0 to 4: one point is, respectively, allocated for the presence of lacunes, microbleeds, moderate to severe (>10) PVS in basal ganglia, periventricular WMH Fazekas 3 or deep WMH Fazekas 2–3.

### 3.2 Inter- and intra-rater agreements

The inter-rater agreement for ground truth masks was excellent, with *κ* = 0.89 and Dice = 0.83. The intra-rater agreement was excellent in the follow-up assessment, with κ = 0.91 and ICC = 0.91.

### 3.3 Quantitative segmentation evaluation

The average automatic segmentation computation time per slice was 105.3 msec. The results of multiple methods on the four CSVD segmentations on the internal and external datasets are shown in Tables 2, 3. Overall, the proposed model was in agreement with both datasets. All of the CSVD markers had high accuracy and specificity because of the small proportion between the focal area of small vessel disease and overall slices. For the internal dataset, the specificity results of WMH, CMBs, lacune, and EPVSs were 82.14%, 75.20%, 82.52%, and 74.40%, respectively; the precision results were 87.86%, 73.28%, 69.88%, and 75.99%, respectively, and the HD95 results were 2.4, 4.2, 3.5, and 4.1, respectively. More importantly, the Dice coefficients of the four markers in the internal data were 0.85, 0.74, 0.76, and 0.75, respectively. We have also tested the per-lesion sensitivity, with the results were 69.8%, 72.5%, and 70.2% for CMBs, lacune, and EPVSs, respectively. Compared with the other methods, especially for the most challenging segmentations of EPVSs, the proposed method showed more than a 10% improvement in the Dice coefficients. Overall, the proposed method had higher accuracy in all of the evaluation metrics.

**Table 2:** Quantitative evaluation of segmentation results by the proposed method with other comparison methods on the internal dataset.

CSVD markers	Method	Pre (95% CI)	Sp (95% CI)	Dice (95% CI)	Per-lesion Sen (95% CI)	HD95 (mm, 95% CI)	*p*-value
WMH	UNET	77.8% (74.2–81.3)	78.8% (75.1–82.3)	0.78 (0.75–0.81)	/	3.5 (3.1–3.9)	/
	Res-NET	83.3% (80.1–86.4)	78.1% (74.5–81.6)	0.81 (0.78–0.84)	/	2.9 (2.5–3.3)	**
	DeepLabV3	83.5% (80.3–86.6)	77.2% (73.6–80.7)	0.80 (0.77–0.83)	/	3.0 (2.6–3.4)	**
	Proposed	87.9% (85.2–90.5)	82.1% (78.8–85.4)	0.85 (0.83–0.87)	/	2.4 (2.0–2.8)	***
CMBs	UNET	64.2% (59.7–68.6)	67.8% (63.3–72.2)	0.66 (0.62–0.70)	54.3% (49.8–58.7)	5.8 (5.2–6.4)	/
	Res-NET	67.7% (63.3–72.0)	69.4% (65.0–73.7)	0.69 (0.65–0.73)	58.9% (54.5–63.2)	5.2 (4.7–5.7)	*
	DeepLabV3	70.8% (66.5–75.0)	69.5% (65.1–73.8)	0.70 (0.66–0.74)	61.2% (56.8–65.5)	5.0 (4.5–5.5)	*
	Proposed	73.3% (69.1–77.4)	75.2% (71.0–79.3)	0.74 (0.70–0.78)	69.8% (65.6–73.9)	4.2 (3.7–4.7)	**
Lacune	UNET	62.4% (58.0–66.7)	76.4% (72.3–80.4)	0.69 (0.65–0.73)	62.1% (57.7–66.4)	4.5 (4.0–5.0)	/
	Res-NET	65.7% (61.3–70.0)	78.9% (74.9–82.8)	0.72 (0.68–0.76)	65.7% (61.4–69.9)	4.1 (3.6–4.6)	**
	DeepLabV3	67.2% (62.9–71.4)	80.1% (75.1–83.0)	0.73 (0.69–0.77)	67.2% (63.0–71.3)	4.0 (3.5–4.5)	**
	Proposed	69.9% (65.7–74.0)	82.5% (78.8–86.1)	0.76 (0.72–0.80)	72.5% (68.4–76.5)	3.5 (3.0–4.0)	***
EPVSs	UNET	58.6% (53.9–63.2)	59.4% (54.7–64.0)	0.59 (0.54–0.64)	51.8% (47.2–56.3)	6.2 (5.6–6.8)	/
	Res-NET	61.2% (56.6–65.7)	63.1% (58.5–67.6)	0.62 (0.57–0.67)	55.3% (50.7–59.8)	5.7 (5.1–6.3)	**
	DeepLabV3	65.9% (61.4–70.3)	64.4% (59.9–68.8)	0.65 (0.60–0.70)	59.1% (54.6–63.5)	5.3 (4.8–5.8)	***
	Proposed	76.0% (71.9–80.0)	74.4% (69.8–78.1)	0.75 (0.71–0.79)	70.2% (66.0–74.3)	4.1 (3.6–4.6)	***

WMH, white matter hyperintensity; CMBs, cerebral microbleeds; EPVSs, enlarged perivascular spaces; Pre, precision; Sp, specificity; Sen, sensitivity; HD95, Hausdorff distance; ^*^p < 0.05; ^**^p < 0.01; ^***^p < 0.001.

**Table 3:** Quantitative evaluation of segmentation results by the proposed method with other comparison methods on the external dataset.

CSVD markers	Method	Pre (95% CI)	Sp (95% CI)	Dice (95% CI)	Per-lesion Sen (95% CI)	HD95 (mm, 95% CI)	*p*-value
WMH	UNET	71.6% (66.5–76.6)	58.5% (53.2–63.7)	0.64 (0.59–0.69)	/	4.8 (4.2–5.4)	/
	Res-NET	75.3% (70.4–80.1)	65.6% (60.4–70.7)	0.70 (0.65–0.75)	/	4.1 (3.6–4.6)	**
	DeepLabV3	75.2% (70.3–80.1)	67.7% (62.6–72.7)	0.71 (0.66–0.76)	/	3.9 (3.4–4.4)	***
	Proposed	86.0% (82.2–89.8)	70.9% (66.0–75.8)	0.78 (0.74–0.82)	/	3.1 (2.6–3.6)	***
CMBs	UNET	50.8% (45.1–56.5)	55.7% (50.0–61.4)	0.53 (0.47–0.59)	48.3% (42.8–53.8)	7.2 (6.5–7.9)	/
	Res-NET	55.8% (50.1–61.5)	60.2% (54.6–65.7)	0.58 (0.52–0.64)	53.7% (48.1–59.3)	6.5 (5.9–7.1)	*
	DeepLabV3	56.3% (50.6–62.0)	60.4% (54.8–66.0)	0.58 (0.52–0.64)	54.2% (48.6–59.8)	6.3 (5.7–6.9)	*
	Proposed	62.5% (56.9–68.1)	65.1% (59.6–70.6)	0.64 (0.58–0.70)	61.9% (56.5–67.3)	5.4 (4.8–6.0)	**
Lacune	UNET	58.9% (53.2–64.6)	60.2% (54.5–65.9)	0.60 (0.54–0.66)	57.4% (51.8–63.0)	5.5 (4.9–6.1)	/
	Res-NET	68.2% (62.7–73.7)	66.1% (60.6–71.6)	0.67 (0.61–0.73)	65.2% (59.7–70.7)	4.7 (4.1–5.3)	**
	DeepLabV3	67.3% (61.8–72.8)	67.8% (62.3–73.3)	0.66 (0.60–0.72)	63.8% (58.2–69.4)	4.9 (4.3–5.5)	**
	Proposed	80.4% (75.6–85.2)	74.1% (68.9–79.3)	0.77 (0.72–0.82)	76.5% (71.5–81.5)	3.8 (3.3–4.3)	***
EPVSs	UNET	55.3% (49.5–61.1)	41.5% (36.0–47.4)	0.47 (0.41–0.53)	43.1% (37.5–48.7)	8.1 (7.3–8.9)	/
	Res-NET	61.9% (56.2–67.6)	49.2% (43.4–55.0)	0.55 (0.49–0.61)	51.6% (45.9–57.3)	7.3 (6.6–8.0)	**
	DeepLabV3	62.7% (57.0–68.4)	50.3% (44.6–56.0)	0.56 (0.50–0.62)	52.8% (47.1–58.5)	7.1 (6.4–7.8)	***
	Proposed	84.8% (80.4–89.2)	64.0% (58.4–69.6)	0.73 (0.68–0.78)	68.9% (63.6–74.2)	5.2 (4.6–5.8)	***

WMH, white matter hyperintensity; CMBs, cerebral microbleeds; EPVSs, enlarged perivascular spaces; Pre, precision; Sp, specificity; Sen, sensitivity; HD95, Hausdorff distance; ^*^p < 0.05; ^**^p < 0.01; ^***^p < 0.001.

Additionally, the robustness of each method was tested via external datasets. Owing to the data bias from different equipment, most of the results on the external dataset were degraded to some extent. Nevertheless, the proposed method also had the lowest decrease when compared with the other methods. Specifically, the Dice values of UNET, Res-NET, DeepLabV3, and the proposed method decreased by 0.12, 0.085, 0.093, and 0.045 between the internal and external data. The smaller degradation reflected a stronger generalization ability of the proposed method, especially on the lacune and EPVS, which were more difficult to discriminate in clinical practice in some instances.

**Figure 3:**
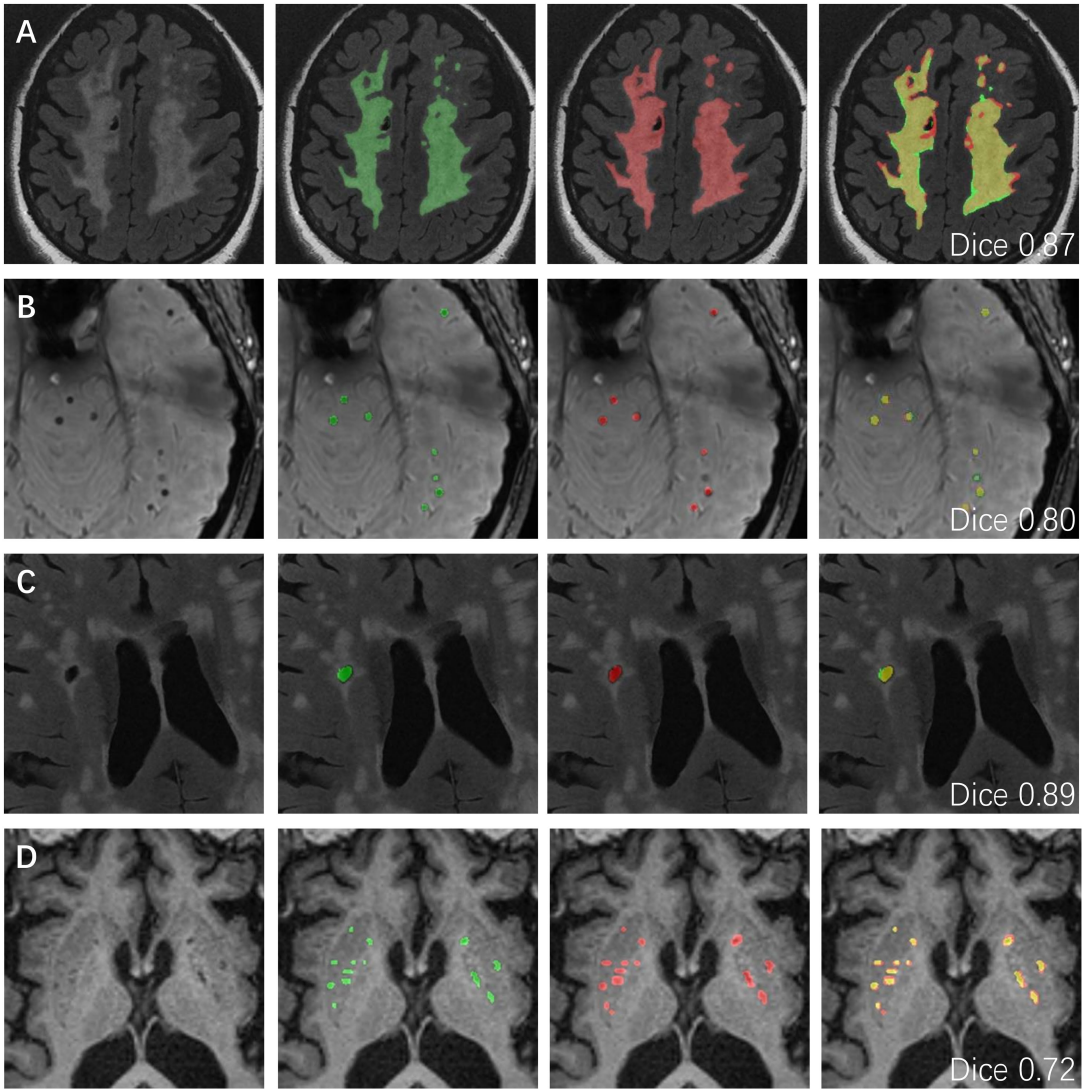
The representative ground truth (in green) and automated segmentation (in red) images of CSVD imaging markers in the internal data set. The differences between manual labeling and DL-based segmentation are highlighted in yellow. **(A)** WMH segmentation results from a 50-year-old female with CSVD. The dice value is 0.87. **(B)** CMBs segmentation results from a 62-year-old male with a dice value of 0.80. **(C)** Lacune segmentation results from a 56-year-old male with a dice value of 0.89. **(D)** EPVSs segmentation results from a 75-year-old male with a dice value of 0.72. CSVD, cerebral small vessel disease; WMH, white matter hyperintensity; CMBs, cerebral microbleeds; EPVSs, enlarged perivascular spaces; DL, deep learning.

**Figure 4:**
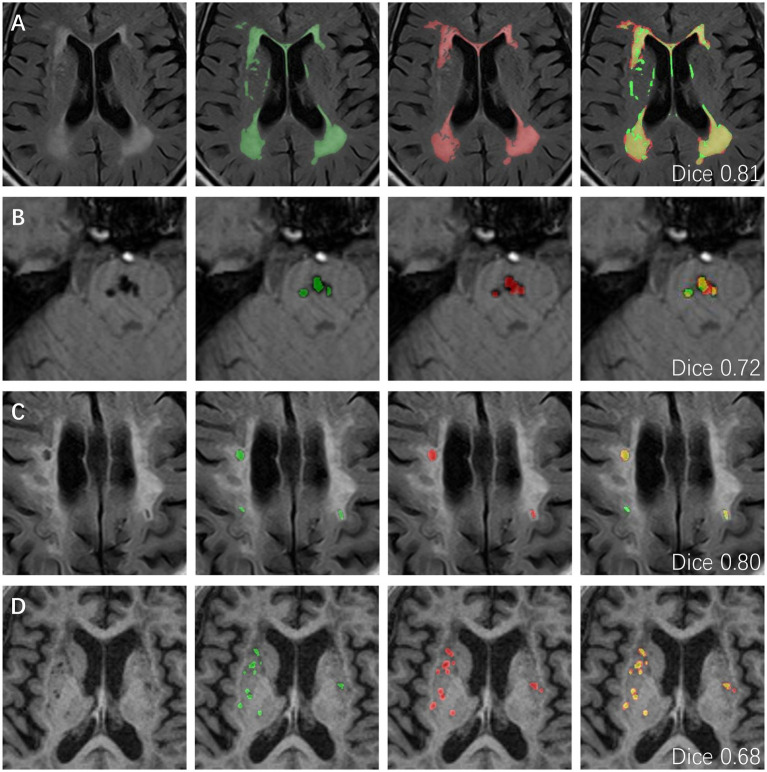
The representative ground truth (in green) and automated segmentation (in red) images of CSVD imaging markers in the external data set. The differences between manual labeling and DL-based segmentation are highlighted in yellow. **(A)** WMH segmentation results from a 74-year-old male with a dice value of 0.81. **(B)** CMBs segmentation results from a 65-year-old male with a dice value of 0.72. **(C)** Lacune segmentation results from a 76-year-old male with a dice value of 0.80. **(D)** EPVSs segmentation results from a 66-year-old female with a dice value of 0.68. CSVD, cerebral small vessel disease; WMH, white matter hyperintensity; CMBs, cerebral microbleeds; EPVSs, enlarged perivascular spaces; DL, deep learning.

Representative visual examples of the four CSVD markers and the corresponding Dice values for both the internal and the external datasets are shown in Figures 3, 4. Although some controversies remain regarding some fuzzy lesions, good consistency can be achieved between the ground truth and automated segmentation for most lesions.

### 3.4 Agreement in calculated values between DL and manual approaches

**Figure 5:**
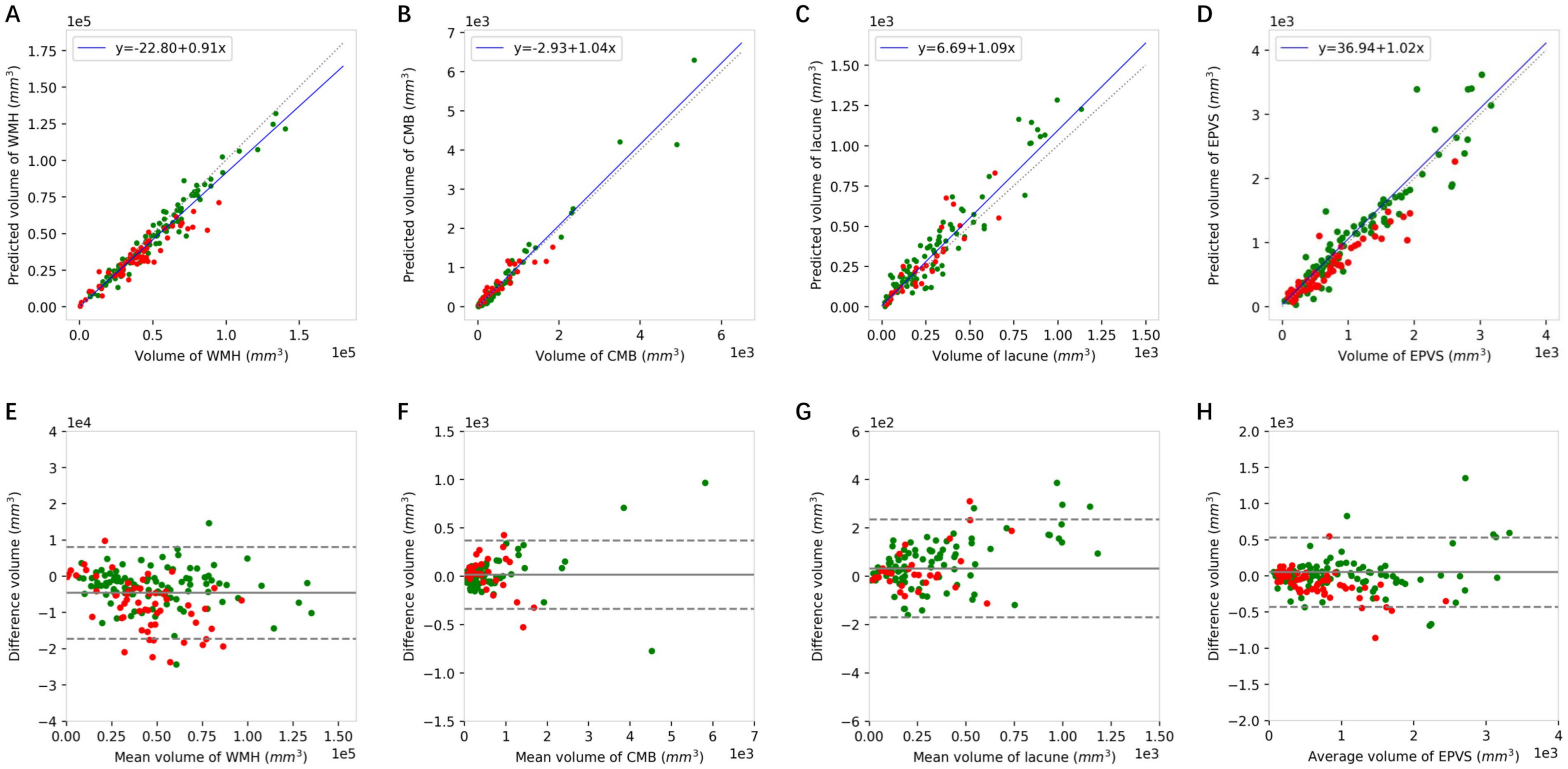
The Pearson correlation **(A–D)** and Bland–Altman analysis **(E–H)** of WMH, CMBs, lacune, and EPVSs between the volumes quantified using the DL model and the corresponding volumes of the ground truth. Green dots represent the results of the internal dataset and red dots represent the results of the external dataset. WMH, white matter hyperintensity; CMBs, cerebral microbleeds; EPVSs, enlarged perivascular spaces.

The volume of each CSVD marker can be obtained by multiplying the mask of each of the four markers and the 3D voxel spacing of the different MRI sequences. Then, the correlation between the results obtained via clinical annotation and DL can be analyzed. The results of the Pearson correlation analyses are shown in Figures 5a–d and the detailed correlation coefficients are listed in Table 4. With all Pearson correlation coefficients greater than 0.90, the results reflected a positive correlation and a high degree of reproducibility. Specifically, the overall Pearson correlations of WMH, CMBs, lacune, and EPVSs were 0.968, 0.978, 0.948, and 0.947, respectively. The Bland–Altman plot in Figures 5e–h also shows good accordance as well.

**Table 4:** Correlation performance between the DL model and manual approach.

CSVD markers	Pearson correlation coefficients		
	Internal	External	Overall
WMH	0.981^***^	0.945^***^	0.968^***^
CMBs	0.984^***^	0.917^***^	0.978^***^
Lacune	0.953^***^	0.906^***^	0.948^***^
EPVSs	0.952^***^	0.945^***^	0.947^***^

WMH, white matter hyperintensity; CMBs, cerebral microbleeds; EPVSs, enlarged perivascular spaces. ^**^p < 0.001.

### 3.5 Differences in volumes among the respective visual scores of different neuroimaging markers

**Figure 6:**
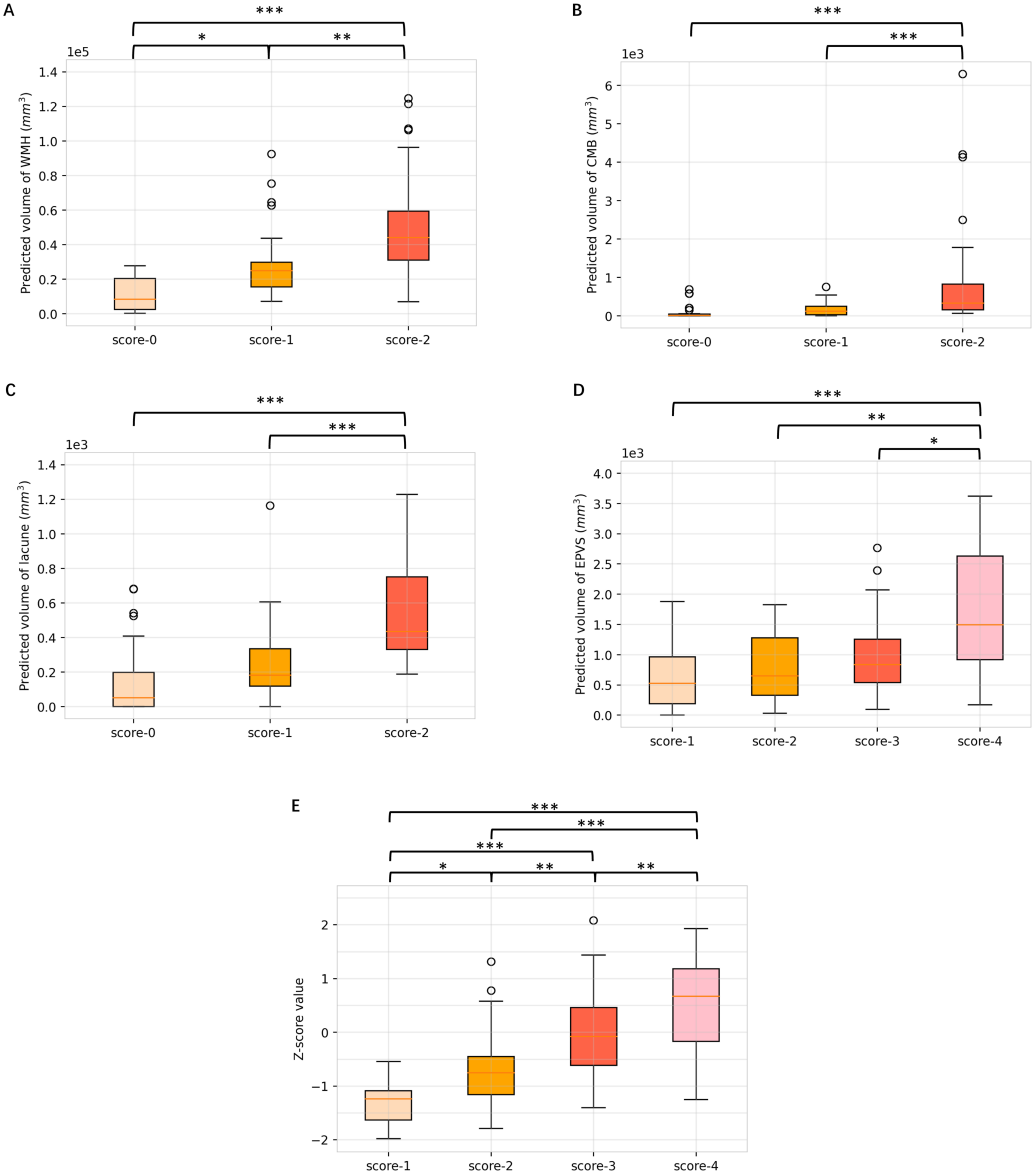
Box plots of differences in volumes among respective visual scores of WMH, CMBs, lacune, EPVSs, and total burden. **(A)** WMH; **(B)** CMBs; **(C)** lacune; **(D)** EPVSs; **(E)** total CSVD burden. Multiple comparison correction was performed using the Least Significant Difference (LSD). ^*^p < 0.05; ^**^p < 0.01; ^***^p < 0.001.

As shown in Figure 6, there were substantial differences in the WMH volumes according to DL segmentations among different visual scores of WMH (*p* < 0.001), and WMH volumes were significantly different between all pairs of scores. In addition, the quantitative volumes of lacunes and CMBs increased accordingly as the qualitative visual score increased from low to high (*p* < 0.001). As for EPVSs, there was an increasing trend in the EPVSs volume as the visual score increased (*p* < 0.001); however, significance only existed in the comparisons between scores of 4 and other scores. ANOVA revealed significant differences in Z scores among patients with different total burden scores (*p* < 0.001). Additionally, post-hoc analysis revealed significant differences in almost all pairs of scores, as shown in Supplementary Table S1.

## 4 Discussion

In this work, a DL model was built for accurate segmentation of four neuroimaging markers of CSVD that could help clinicians obtain a precise diagnosis of the disease. To the best of our knowledge, this is the first DL architecture designed for the simultaneous segmentation of four markers of CSVD in multisequence MRI. Over the 105 subjects in the internal datasets, the Dice values of WMH, CMBs, lacune, and EPVSs achieved 0.85, 0.74, 0.76, and 0.75. The proposed model also obtained high accuracy and consistency compared with the gold standard lesion volume obtained by clinicians. Furthermore, the quantitative Z scores generated by the model reflects the CSVD global burden that correlated well with the widely recognized total burden score.

In current clinical practice, the diagnosis of CSVD relies primarily on neuroimaging features. To date, quantitative and accurate diagnosis remains challenging (3). Various visual rating scores have been developed to simply stratify the severity of CSVD and have assisted in the statistical analysis of data (6, 33). Nonetheless, these scores have not achieved full generalizability, and significant heterogeneity may exist in total CSVD scores determined by different doctors for the same patient. Moreover, visual composite scores are less sensitive in accurately detecting global brain changes. Owing to the rapid progression of DL technology, efficient and accurate segmentation has been accomplished in numerous medical imaging scenarios. For the segmentation of CSVD, most previous works focused on WMH (15–20, 34). In addition, other works have focused on CMBs (21, 35) and lacunes (22). However, CSVD is composed of multiple lesions and requires different MRI sequences for diagnosis. Further, the lesions associated with CSVD are more insidious, numerous, and varying than those associated with other diseases. Thus, the results of commonly used models or large-scale medical segmentation models (36, 37) are unsatisfactory. A recent study investigated the link between cognitive outcomes and automated MRI segmentation features of multiple types of CSVD-related brain changes (14); nevertheless, CMBs were not included in the analyses, despite being typical CSVD neuroimaging features (38).

In this work, we developed a deep learning model for simultaneous segmentation of four CSVD neuroimaging markers across multi-sequence MRI, advancing beyond prior single-marker approaches. Owing to the specificity of medical imaging, direct migration pretrained parameters such as ImageNet (39) are unsatisfactory. Furthermore, the relative scarcity of medical imaging data leads to overfitting when each CSVD marker is trained. We adopted two approaches to solve the above problem. First, we pre-trained the raw data of the four CSVD markers by an auto-encoder network and migrated the parameters of the encoder part to the segmentation network. Pretraining the model using contrastive learning on unannotated multi-sequence MRI data enabled robust feature extraction by learning anatomical consistency across modalities and vascular pattern representations. This approach mitigated data scarcity constraints, enhanced cross-sequence alignment, and improved small lesion detection sensitivity. Second, owing to the structural similarity and specificity of brain images from different sequences, we designed a network with a shared encoder block and four separate decoder blocks. Unlike conventional multi-model pipelines, our design employs a shared encoder with cross-sequence attention mechanisms that explicitly model anatomical coherence. For example, the MRI characteristic of the perivascular space in T1 is aligned with that of lacune, which has a central hypointensity with a surrounding rim of hyperintensity in FLAIR. Meanwhile, each lesion corresponding to its respective decoder had the ability to capture different lesion features, such as CMBs with specific hypointensity in SWI but with iso-intense signal in other sequences. In this study, we also conducted experiments with five-fold cross-validation and tested the generalizability of the model on a dataset of different equipment. The obtained results strongly support the validity and generalizability of the designed model, as it outperforms the comparison methods on both datasets.

The model in our study is one of the few comprehensive quantitative evaluations of the total CSVD imaging burden. Our results suggested that automated segmentation based on the current DL model could achieve good concordance with manual delineation. The quantitative volumes of CSVD markers and Z scores correlated well with the corresponding visual scores, except for EPVSs. We propose that this DL algorithm has advantages in enabling a more rapid, accurate, and homogeneous diagnosis of CSVD burden and facilitating promising improvement in the diagnosis of CSVD from the existing qualitative evaluation to a more refined quantitative diagnosis. However, the association with clinical performance still needs further study. Notably, the differences in the segmented EPVS volumes among the groups with different visual scores were not as significant as those among the other groups. The potential reasons may be as follows: First, the sensitivity of EPVS segmentation based on the 3D-T1 sequence is relatively lower than that of the qualitative score based on the T2 sequence (40). Second, neurologists and radiologists can identify vague EPVSs on 3D-T1 images, which the DL model may inevitably neglect. Moreover, the evaluation of EPVSs severity in the existing total burden score is commonly based on the number of EPVSs in the basal ganglia (6), whereas our delineation of EPVSs focused mainly on the whole brain. Further studies are needed to assess the correlation between the EPVS volume in different regions predicted by DL and the development of CSVD.

While our model demonstrates promising performance, several limitations merit careful consideration. First, the single ethnic cohort and hospital-based data may limit generalizability to populations with diverse demographics or other 1.5 T/7 T imaging configurations and introduce bias toward severe phenotypes, thereby compromising the early disease detection performance of the method. Then, while transfer learning was partially addressed via pretraining, domain adaptation techniques like adversarial feature alignment were not explored to mitigate scanner-specific intensity variations, which contributed to external validation performance. Moreover, owing to the low incidence of recent subcortical infarcts and cortical microinfarcts in the two datasets, we could not include these two markers in our analyses. Finally, the current quantitative results focus only on the volume of the CSVD markers. More in-depth details, such as the number, location, and size of lesions, as well as a more comprehensive method that includes cerebral atrophy need to be considered to achieve a more precise diagnosis.

## 5 Conclusion

In conclusion, a DL model for the segmentation of four CSVD neuroimaging markers was developed, which has high spatial accuracy and volumetric consistency with manual annotation. This quantitative evaluation tool enables the clinical judgment of CSVD from qualitative analysis to quantitative diagnosis. Future research will focus on the clinical impact of morphology and location of different lesions, leading to a more refined and personalized diagnosis of CSVD.

## Data Availability

The original contributions presented in the study are included in the article/Supplementary material, further inquiries can be directed to the corresponding authors. Requests to access these datasets should be directed to JF, jianhuifu@126.com.

## Footnotes

^1^ https://www.python.org

^2^ https://pytorch.org
