# Supplementary material for: Deep learning-based automated segmentation for the quantitative diagnosis of cerebral small vessel disease via multisequence MRI
Source: Front Neurol. 2025 May 27;16:1540923. doi: 10.3389/fneur.2025.1540923 (PMC12150800; doi:10.3389/fneur.2025.1540923)
Supplement: Supplementary file 1 [file Data_Sheet_1.docx]

**Inclusion and exclusion criteria:**

The full inclusion and exclusion criteria for the two datasets have been published previously[Discovery medicine. 2017;23(126):175-82 / Eur Radiol. 2023;33(9):6096-106].

The inclusion criteria as follows: 1) moderate to severe white matter hyperintensity(Fazekas score of 2-3) and at least one of the three characteristic markers(lacune, microbleeds, and enlarged perivascular spaces) and 2)aged 50-80 years old.

The exclusion criteria were: 1)cerebral infarction with a diameter of infarction core larger than 2cm; 2)cortical infarction; 3)stroke due to any potential cardioembolic source; 4)lacunar stroke syndrome within 6 months; 5)cerebrovascular stenosis with >50% luminal stenosis suggested by transcranial doppler ultrasound, computed tomography angiography or magnetic resonance angiography; 6)genetically confirmed hereditary CSVD or suspected hereditary CSVD according to the family history and clinical features; 7)potential inflammatory and immunologically mediated CSVD according to clinical features and immune index; 8)white matter diseases that might be caused by other reasons except CSVD, such as multiple sclerosis; 9)cognitive impairment that might be caused by other diseases, such as neurodegeneration disease or hydrocephalus; 10)severe depression or other affective disorder that affect cognitive evaluation; 11)language disturbances or severe affective disturbance that impairs communication; and 12)MRI contraindications.
