# Supplementary material for: Deep learning-based automated segmentation for the quantitative diagnosis of cerebral small vessel disease via multisequence MRI
Source: Front Neurol. 2025 May 27;16:1540923. doi: 10.3389/fneur.2025.1540923 (PMC12150800; doi:10.3389/fneur.2025.1540923)
Supplement: Supplementary file 2 [file Table_1.docx]

**Supplemental Materials**

**Table S1. The raw lesion volumes(mm^3^) in CSVD subjects with different visual scores**

| Visual scores | WMH^*^ | | CMB^**^ | | Lacune^§^ | | EPVS^¶^ | | Z score^‡^ | |
| --- | --- | --- | --- | --- | --- | --- | --- | --- | --- | --- |
|  | **N** | **volume** | **N** | **volume** | **N** | **volume** | **N** | **volume** | **N** | **value** |
| **Score 0** | 10 | 11899.75 | 47 | 92.87 | 65 | 137.65 | / | / | / | / |
| **Score 1** | 48 | 33608.87 | 45 | 173.84 | 61 | 238.48 | 38 | 673.96 | 6 | -1.30 |
| **Score 2** | 105 | 45502.31 | 71 | 746.76 | 37 | 558.36 | 56 | 726.15 | 23 | -0.59 |
| **Score 3** | / | / | / | / | / | / | 45 | 978.02 | 34 | 0.05 |
| **Score 4** | / | / | / | / | / | / | 24 | 1820.94 | 56 | 0.51 |

Abbreviations: CSVD, cerebral small vessel disease; WMH, white matter hyperintensity; CMBs, cerebral microbleeds; EPVSs, enlarged perivascular spaces

* WMH were graded according to the sum of deep and periventricular WMH Fazekas scale(0 to 3): 1= total periventricular+ subcortical WMH grade 3–4; 2= grade 5-6.

**CMBs were rated 1 for 1-10 CMBs and rated 2 for >10 CMBs.

§ Lacunes were rated 0 if none was present, rated 1 for 1-4 lacunes and rated 2 for >4 lacunes.

¶ The categories of EPVSs are as follows: 0=none, 1=1-10, 2=11-20, 3=21-40, 4=>40 EPVSs in the basal ganglia.

‡ The visual score of total CSVD burden ranges from 0 to 4: one point is respectively allocated for the presence of lacunes, microbleeds, moderate to severe (>10) PVS in basal ganglia, periventricular WMH Fazekas 3 or deep WMH Fazekas 2–3.
